# Trained immunity in inflammatory bone disease: a bibliometric and literature-level text-mining analysis

**DOI:** 10.3389/fimmu.2026.1832996

**Published:** 2026-05-20

**Authors:** Zihang Zhao, Zihan Liu, Liang Guo, Teng Pan, Ruoyu Wang, Yiran Li, Yingchao Yin, Ruipeng Zhang, Zhiyong Hou

**Affiliations:** 1 Department of Orthopaedic Surgery, Third Hospital of Hebei Medical University, Shijiazhuang, China; 2 Hebei Medical University, Shijiazhuang, China; 3 Department of Oncology, Shijiazhuang People’s Hospital, Shijiazhuang, China

**Keywords:** epigenetic reprogramming, inflammatory bone disease, innate immune memory, myelopoiesis, osteoclastogenesis, osteoimmunology, periodontitis, trained immunity

## Abstract

**Background:**

Trained immunity provides a framework for understanding persistent innate immune reprogramming in chronic inflammatory and immune-mediated disorders. Its relationship with osteoimmunology and inflammatory bone disease, however, remains insufficiently characterized.

**Methods:**

We performed a bibliometric and literature-level text-mining analysis using WoSCC as the primary source for formal bibliometric analyses. A two-corpus design was used, with a core corpus for disease-focused bibliometric analysis and an extended corpus for semantic screening and sensitivity analyses. Scopus and PubMed were queried independently for cross-database comparison. Topic modeling, TF-IDF-based semantic screening, disease-context analysis, and bridge-network mapping were used to summarize literature-level patterns.

**Results:**

The core corpus included 83 records, and the extended corpus included 301 records. Within the full-year 2013–2025 comparison window, publication output increased over time and reached its highest level in 2025. Cross-database comparison showed broadly concordant publication trends and substantial DOI-level overlap across WoSCC, Scopus, and PubMed. Periodontitis was the most prominent disease context, whereas osteoarthritis, ankylosing spondylitis, osteoporosis, and inflammatory arthritis also appeared in the disease-context mapping. Across bibliometric, semantic, and mapping analyses, recurrent literature-level patterns involved myeloid-cell reprogramming, epigenetic and metabolic remodeling, macrophage- and monocyte-associated terms, myelopoiesis, innate immune signaling, and osteoclast-related pathways.

**Conclusion:**

Research on trained immunity in inflammatory bone disease remains relatively small but is expanding. Current literature most consistently connects trained-immunity-related concepts with myeloid reprogramming, immunometabolic and epigenetic processes, periodontitis, and inflammatory bone remodeling. These findings provide a structured literature-level overview of an emerging field and may help guide future mechanistic, experimental, and translational studies.

## 1 Introduction

Trained immunity is increasingly discussed as a conceptual framework for understanding how innate immune cells acquire memory-like programs that shape chronic inflammation, tissue remodeling, and disease susceptibility (1–4). Unlike classical adaptive immune memory, trained immunity is driven mainly by epigenetic remodeling and metabolic rewiring, and can involve not only mature innate immune cells but also hematopoietic stem and progenitor compartments (1–6). Initially emphasized in infection and vaccination biology, this concept is now increasingly being extended to chronic inflammatory and immune-mediated diseases (2–4).

These developments are particularly relevant to osteoimmunology, which describes the reciprocal regulation between the immune system and skeletal homeostasis (7). In inflammatory bone disorders, immune-cell activation is tightly linked to osteoclastogenesis, impaired bone formation, and pathological remodeling, with monocytes/macrophages, neutrophils, cytokine networks, and bone marrow-derived progenitors all contributing to disease progression (7, 8). Within this context, trained immunity provides a biologically plausible framework for understanding how persistent innate immune reprogramming may intersect with inflammatory bone loss.

Several disease settings illustrate this potential link. Periodontitis has emerged as a particularly relevant model because it combines chronic microbial stimulation, dysregulated innate immunity, cytokine-driven tissue destruction, and alveolar bone loss, while also showing links to systemic inflammatory comorbidities (9–11). Parallel lines of research in inflammatory arthritis, osteoarthritis, ankylosing spondylitis, and osteoporosis further support the importance of immune–bone crosstalk in chronic skeletal disease (12–15). Together, these observations suggest that trained immunity may be relevant across multiple inflammatory bone disease contexts rather than being confined to a single disease silo.

Despite this growing interest, the literature at the intersection of trained immunity and osteoimmunology remains fragmented across periodontal disease, rheumatic inflammation, degenerative joint disease, osteoporosis, and related inflammatory bone conditions. As a result, this emerging field remains insufficiently characterized in terms of publication patterns, thematic concentration, intellectual foundations, and disease-related research emphasis. It is also still unclear how innate immune memory is being positioned within the broader inflammatory bone-loss literature rather than within isolated disease-specific contexts. To address this gap, we performed a bibliometric and literature-level text-mining analysis using a two-corpus design that integrated conventional bibliometric analyses with semantic expansion, topic modeling, and multi-layer entity, pathway, and disease mapping. Our aim was not to infer causality, but to characterize how trained-immunity-associated concepts, cells, pathways, and disease contexts are represented across the osteoimmunology literature and connected at the literature level within inflammatory bone disease research.

## 2 Materials and methods

### 2.1 Data source and study design

This study was designed as a systematic search-based bibliometric and literature-level text-mining analysis of trained immunity in osteoimmunology and inflammatory bone disease. Reporting was organized with reference to the PRISMA-ScR framework because the objective was to map the scope, structure, and thematic organization of an emerging research area (16).

A two-corpus design was used to balance disease specificity with analytical breadth. The core corpus served as the main disease-focused dataset for bibliometric analyses, whereas the extended corpus was used to examine a broader trained-immunity-related bone literature through semantic screening and sensitivity analyses. WoSCC was selected as the primary source because it provides structured citation metadata and cited-reference records compatible with established science-mapping workflows implemented in bibliometrix (17). Scopus and PubMed were queried independently to examine whether the main literature-level patterns were consistent across databases with different indexing profiles.

**Figure 1:**
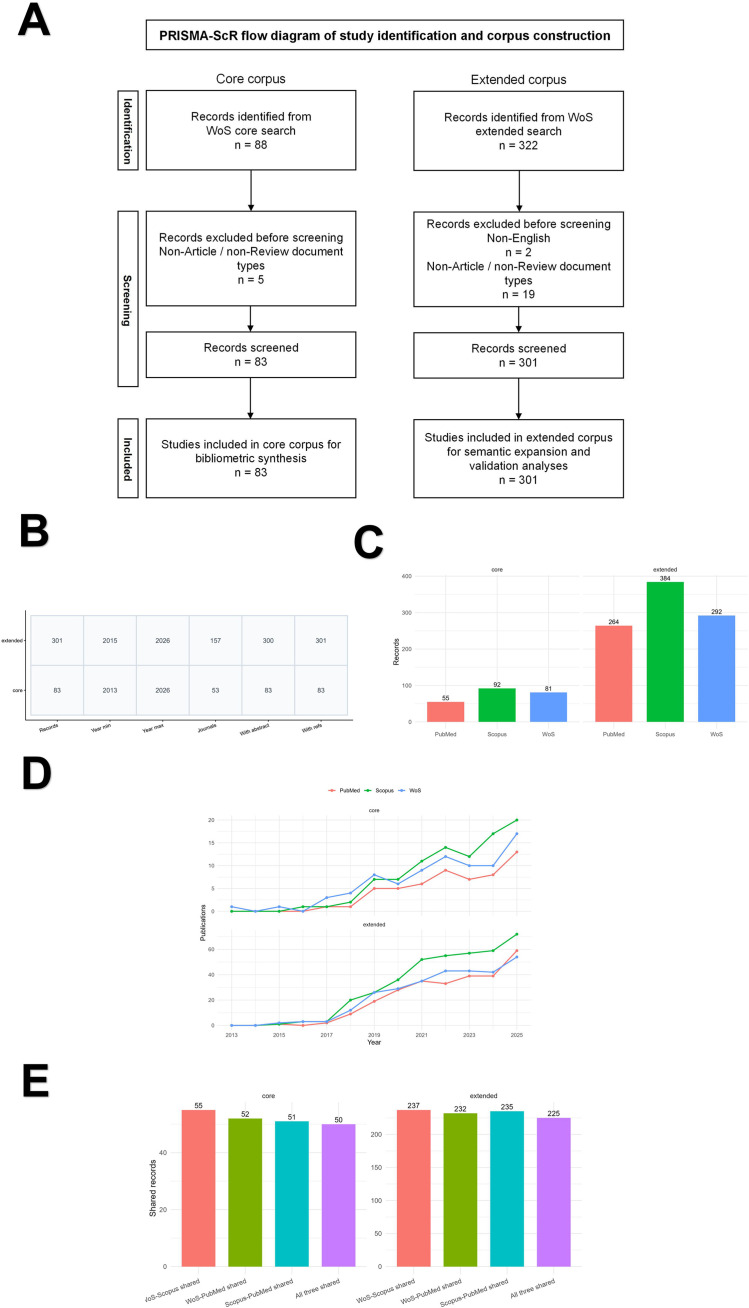
Study design, primary dataset characteristics, and independent multi-database queries. **(A)** PRISMA-ScR flow diagram showing study identification and corpus construction for the core and extended Web of Science Core Collection (WoSCC) datasets. WoSCC was retained as the primary source for formal bibliometric analyses, whereas Scopus and PubMed were queried independently for cross-database comparison. **(B)** Summary metrics of the retained WoSCC primary datasets. **(C)** Sizes of the database-specific query sets used for cross-database comparison within the 2013–2025 window. **(D)** Annual publication trends across WoSCC, Scopus, and PubMed for the core and extended query layers. **(E)** DOI-level overlap across database-specific query sets. Comparative analyses across databases were restricted to 2013–2025 because 2026 was an incomplete publication year at the time of retrieval. Cross-database thematic concordance for selected anchor terms is shown in **Supplementary Figure 1**, and the underlying anchor-term frequencies and proportions are summarized in **Supplementary Table 8**. WoS denotes the Web of Science Core Collection (WoSCC).

The multi-database component was intended for cross-database comparison rather than for construction of a merged master corpus. Because 2026 was an incomplete publication year at the time of retrieval, cross-database trend comparisons were restricted to 2013–2025. **Figure 1** summarizes study identification, dataset construction, and the independent multi-database comparison. The corresponding **Supplementary Materials** provide detailed reproducibility information and supporting analyses, including supplementary LDA-based topic-model checks, analytical parameters and mapping rules, database-specific search strategies, retrieval and filtering counts, DOI-level overlap, and anchor-term frequencies underlying **Supplementary Figure 1** (**Supplementary Tables 1–8**).

### 2.2 Search strategy

Searches were performed in WoSCC using two Topic-search formulas. The final WoSCC searches were conducted on 2 March 2026, and the final Scopus and PubMed searches were conducted on 17 March 2026. The core search was designed to capture records directly linking trained immunity or innate immune memory with osteoimmunology, bone, joint, periodontal, or inflammatory bone disease terms. The extended search used a broader set of bone- and inflammation-related terms to capture a wider literature periphery for semantic screening.

The core corpus was retrieved using:

(“trained immunity” OR “trained innate immunity” OR “innate immune memory” OR (“innate” NEAR/2 memory) OR (“innate” NEAR/2 training)) AND (oste* OR osteoclast* OR osteoimmun* OR joint* OR cartilage OR “intervertebral disc” OR “bone healing” OR fracture* OR osteolysis OR osteomyelitis OR osteoarthritis OR “rheumatoid arthritis” OR spondyloarthritis OR periodontitis OR osteoporosis)

The extended corpus was retrieved using:

(“trained immunity” OR “trained innate immunity” OR “innate immune memory” OR “trained innate” OR “innate training”) AND (bone OR oste* OR osteoimmun* OR osteoclast* OR “bone marrow” OR arthritis OR periodontitis OR osteoporosis)

Database-adapted versions of these searches were implemented in Scopus and PubMed for independent cross-database comparison. Full database-specific search strings, filters, retrieval dates, and retrieval details are provided in **Supplementary Table 5**.

### 2.3 Eligibility criteria and dataset construction

Only English-language Articles and Reviews were included. The WoSCC core search retrieved 88 records, of which 83 were retained after document-type filtering. The WoSCC extended search retrieved 322 records, of which 301 were retained after exclusion of 2 non-English records and 19 records not indexed as Article or Review. These retained records formed the primary WoS datasets for bibliometric and text-mining analyses.

For Scopus and PubMed, database-adapted searches and equivalent screening logic were applied. To reduce instability from asynchronous database updates and partial-year indexing, cross-database comparisons were restricted to publications from 2013 to 2025. Within this window, the retained query sets included 81 WoS core records, 92 Scopus core records, 55 PubMed core records, 292 WoS extended records, 384 Scopus extended records, and 264 PubMed extended records. Database-specific retrieval and filtering results are summarized in **Supplementary Table 6**.

### 2.4 Cross-database comparison

Scopus and PubMed were queried independently to assess whether the main patterns observed in WoSCC were consistent across databases. The comparison focused on record yield, annual publication trends, DOI-level overlap, and thematic concordance.

Raw record counts were not expected to be identical because the databases differ in coverage, indexing practices, metadata fields, and update schedules. Therefore, we focused on convergence in temporal trends, overlap of DOI-bearing records, and consistency of selected conceptual anchor terms. The database-specific records were not merged into a single master corpus.

### 2.5 Data export and preprocessing

Full WoS records and cited references were exported as the primary input for bibliometric analyses. Scopus and PubMed records were processed separately for cross-database comparison after field harmonization and DOI normalization.

After export, language and document-type restrictions were rechecked. Duplicate records were removed using DOI, accession number where available, and title matching. For text-mining analyses, titles and abstracts were lowercased, cleaned, stemmed, and filtered using a curated stop list. Synonyms were harmonized for trained-immunity-related terms, disease names, institutions, and countries.

### 2.6 Bibliometric analysis

Bibliometric analyses were performed in R using bibliometrix/Biblioshiny workflows, with additional text-processing using quanteda and text2vec. We summarized annual publication output, journals, authors, institutions, countries, keywords, and citation patterns.

Co-authorship analyses were performed at the author, institution, and country levels. Author Keywords and Keywords Plus were analyzed separately to distinguish author-defined terms from database-generated indexing terms. Co-occurrence maps, overlay visualizations, thematic maps, reference co-citation analysis, and burst analyses were used to describe thematic structure, intellectual foundations, and emerging research fronts. Software versions and figure-specific settings are provided in the **Supplementary Methods**.

### 2.7 Topic modeling and semantic screening

Topic modeling and semantic screening were used to summarize recurring themes and evaluate the boundary between the core corpus and the broader extended corpus. For the core corpus, titles and abstracts were used for unsupervised topic modeling with stm in R. Candidate topic numbers from K = 5 to K = 7 were examined, and K = 6 was selected for the main presentation because it provided the clearest interpretable separation of themes. Additional model-selection checks are reported in **Supplementary Tables 1–3**.

For the extended corpus, each record was assigned a TF-IDF cosine relevance score relative to the core corpus. The top 40% of ranked records was used as the default semantically relevant subset. Alternative cutoffs and a rule-based subset were examined as sensitivity analyses. Detailed preprocessing thresholds, scoring definitions, and sensitivity analyses are provided in the **Supplementary Methods** and **Supplementary Table 4**.

### 2.8 Entity, pathway, and disease mapping

A multi-layer text-mining framework was applied to titles, abstracts, and keywords to support literature-level biological interpretation. Four layers were defined before analysis: intervention/stimulus, entity, pathway, and disease context. Terms related to cell types, cellular programs, molecular processes, stimuli, interventions, and diseases were harmonized through synonym normalization and dictionary-based aggregation.

Mapped terms were summarized by document, year, and topic. Disease-context analysis linked topic-associated terms to DisGeNET gene-disease associations through Harmonizome and used Fisher’s exact test with Benjamini-Hochberg correction. Pathway-context analysis used KEGG osteoclast differentiation and selected Reactome pathways related to innate immune signaling, cytokine signaling, neutrophil degranulation, chromatin organization, and metabolism.

These analyses were interpreted as exploratory literature-level mappings. They were not used as direct evidence of causal mechanisms or pathway activation in specific disease models. The data resources, selected pathway anchors, dictionary-based mapping rules, and bridge-network display thresholds are summarized in the **Supplementary Methods** and **Supplementary Table 4**.

### 2.9 Supplementary reproducibility analyses

Detailed analytical parameters are provided in the **Supplementary Methods**. These include software versions, text-preprocessing thresholds, topic-model settings, repeated-run checks, semantic-screening cutoffs, rule-based subset definitions, dictionary-based mapping rules, resource versions, and network display thresholds. These supplementary analyses were used to support reproducibility and to assess whether the main interpretations were robust to alternative topic-model and corpus-boundary settings.

## 3 Results

### 3.1 Dataset construction and overall study flow

**Figure 1** summarizes study identification, dataset construction, and independent multi-database comparison. Under the core search strategy, 88 records were retrieved and 83 records were retained after restriction to the Article and Review document types. Under the extended search strategy, 322 records were retrieved and 301 records were retained after exclusion of 2 non-English records and 19 records not indexed as Article or Review. The final core corpus therefore comprised 83 records, and the final extended corpus comprised 301 records. The core corpus spanned 2013–2026 and covered 53 journals, with complete title, abstract, and cited-reference information available for all included records. The extended corpus spanned 2015–2026, covered 157 journals, and contained abstracts for 300 of 301 records. Because 2026 represented a partial publication year at the time of retrieval, year-based trend analyses were restricted to the 81 core records published between 2013 and 2025, whereas the full screened datasets were retained for citation-based and text-mining analyses. **Figure 1** additionally summarizes the independent multi-database queries used for cross-database comparison, including query-set sizes, annual publication trends, and DOI-level overlap across WoS, Scopus, and PubMed.

### 3.2 Independent multi-database queries and cross-database comparison

Independent multi-database queries identified comparable literature sets across WoS, Scopus, and PubMed within the 2013–2025 comparison window. For the core layer, the resulting query sets comprised 81 WoS records, 92 Scopus records, and 55 PubMed records. For the extended layer, the corresponding query sets comprised 292, 384, and 264 records, respectively (**Figure 1C**). Despite expected differences in database coverage and indexing breadth, annual publication trends were broadly concordant across databases in both the core and extended layers, with clear growth after 2018 and the highest output observed in 2025 (**Figure 1D**).

DOI-level overlap further supported cross-database convergence. In the core layer, 55 records were shared between WoS and Scopus, 52 between WoS and PubMed, 51 between Scopus and PubMed, and 50 across all three databases. In the extended layer, the corresponding overlap counts were 237, 232, 235, and 225, respectively (**Figure 1E**). Detailed DOI-level overlap statistics are provided in **Supplementary Table 7**. Cross-database thematic concordance for selected anchor terms is summarized in **Supplementary Figure 1**, and the underlying anchor-term frequencies and proportions are reported in **Supplementary Table 8**. Together, these results support the robustness of the main literature-level patterns across multiple databases while preserving WoSCC as the primary corpus for formal bibliometric analyses.

### 3.3 Bibliometric landscape of trained immunity in osteoimmunology and inflammatory bone loss

**Figure 2:**
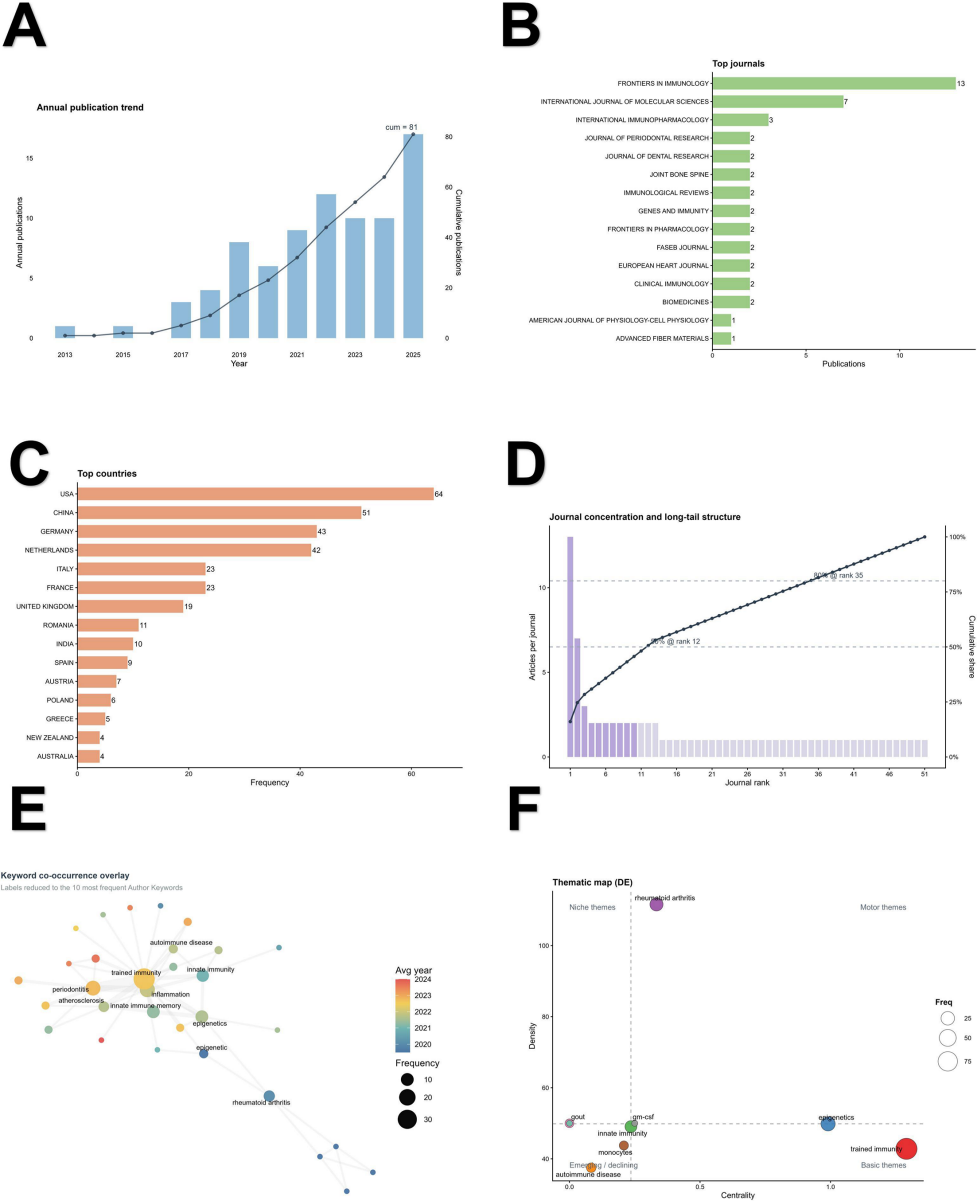
Bibliometric landscape of trained immunity in osteoimmunology and inflammatory bone loss. **(A)** Annual publication trend of the WoSCC primary core corpus from 2013 to 2025, with cumulative growth shown on the secondary axis. **(B)** Top journals by number of publications. **(C)** Top contributing countries by country-occurrence frequency under full counting. **(D)** Journal concentration and long-tail structure, showing the cumulative contribution of ranked journals to the field. **(E)** Author Keyword co-occurrence overlay network. To improve readability, labels were reduced to the most frequent core keywords, whereas low-frequency nodes were retained without labels. Node size indicates keyword frequency, node color represents the average publication year, and edge thickness reflects co-occurrence weight. **(F)** Thematic map of Author Keywords showing the distribution of major conceptual themes according to centrality and density; bubble size reflects keyword frequency.

**Figure 3:**
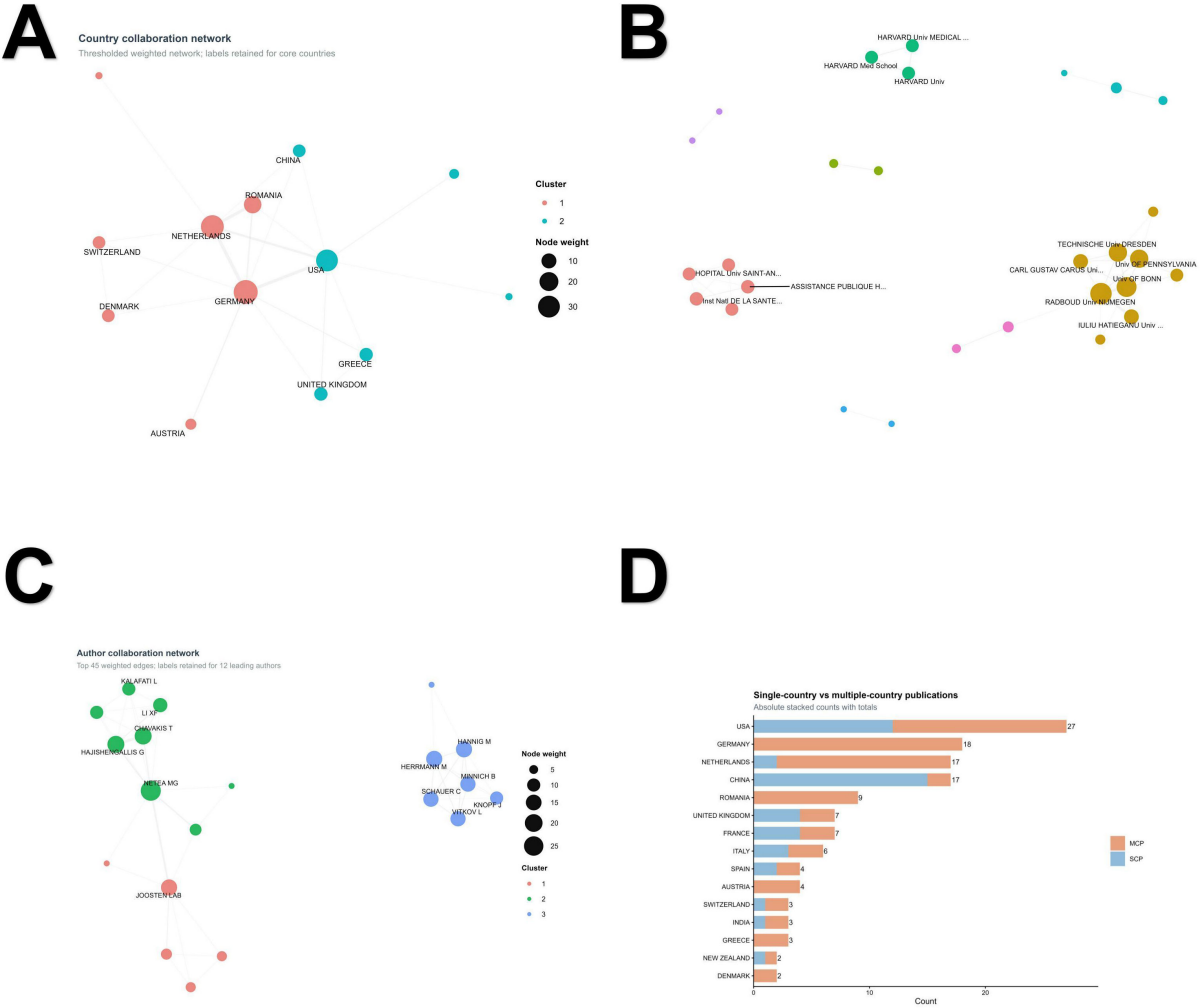
Collaboration structure of the field. **(A)** Country collaboration network based on country-level co-authorship in the core corpus. **(B)** Institution collaboration network displayed as a simplified weighted network. To improve readability, the display was restricted to the top weighted links, and labels were retained only for core high-weight institutions. **(C)** Author collaboration network displayed as a simplified weighted network, with labels retained only for leading authors. In the network panels, node size reflects weighted collaboration strength, edge thickness reflects collaboration weight, and node color indicates network cluster. **(D)** Single-country publications (SCP) and multiple-country publications (MCP) among major contributing countries based on article-level country assignments; these totals are not directly comparable to the country-occurrence counts shown in **Figure 2C**. Detailed network display settings and underlying analytical parameters are provided in the **Supplementary Methods** and **Supplementary Tables**.

A total of 81 core publications published between 2013 and 2025 were included in the year-based bibliometric analyses, showing an overall upward trajectory and reaching a peak annual output of 17 publications in 2025 (**Figure 2A**). Publication activity was concentrated in a limited number of journals. Frontiers in Immunology ranked first with 13 publications, followed by the International Journal of Molecular Sciences with 7 and International Immunopharmacology with 3. Journal concentration analysis further showed that the top journal accounted for 16.0% of the literature, the top 5 journals for 33.3%, and the top 10 journals for 45.7%, while 12 journals were required to reach a 50% cumulative share and 35 journals to reach 80%, indicating a recognizable core-periphery distribution (**Figures 2B, D**). At the country level, the United States, China, and Germany were the leading contributors, with country-occurrence counts of 64, 51, and 43, respectively (**Figure 2C**). These values reflect country appearances under full counting and should be distinguished from the article-level SCP/MCP counts summarized in **Figure 3D**.

Keyword and thematic analyses suggested that the field was organized around a relatively compact but coherent conceptual structure. The Author Keyword overlay network contained 35 nodes and 67 edges, with trained immunity (39), inflammation (16), periodontitis (15), epigenetics (10), and innate immune memory (10) as the most frequent terms. Overlay timing suggested that more recent attention shifted toward hematopoietic stem and progenitor cells, innate immune system, clonal hematopoiesis, and bone and joint infection. The thematic map identified 9 clusters. Trained immunity formed the largest and most central theme (frequency = 97; centrality = 1.29), whereas epigenetics also showed high centrality. Rheumatoid arthritis showed the highest density (111.61), consistent with a specialized and internally cohesive disease-oriented theme. Additional peripheral or niche themes included innate immunity, monocytes, gout, autoimmune disease, GM-CSF, and neutrophil hyper-responsiveness (**Figures 2E, F**).

### 3.4 Collaboration structure and intellectual foundations of the field

Collaboration analyses showed that the field is internationally connected but still organized around a limited number of recurrent partnerships. The country collaboration network included 13 countries and 23 weighted edges. The strongest country-level links were observed between Germany and the Netherlands (weight = 10), the United States and Germany (weight = 9), and the Netherlands and Romania (weight = 9), followed by the United States-Netherlands connection (weight = 7) (**Figure 3A**). At the institutional level, the collaboration network comprised 36 institutions and 61 weighted edges, with the strongest ties centered on Radboud University Nijmegen, the University of Bonn, Technische Universitat Dresden, the University of Pennsylvania, and Iuliu Hatieganu University of Medicine and Pharmacy (**Figure 3B**). At the author level, the network also comprised 36 authors linked by 79 weighted edges and was shaped by recurring collaborations involving Hajishengallis G, Chavakis T, Netea MG, and Riksen NP (**Figure 3C**). Multiple-country publication (MCP) and single-country publication (SCP) analysis, which uses article-level country assignments rather than country-occurrence counts, further showed that the United States had the highest total output (27), while Germany and Romania displayed fully internationalized publication patterns (MCP ratio = 100%). In contrast, China showed a lower MCP ratio of 11.8%, indicating a more domestically concentrated publication pattern (**Figure 3D**).

**Figure 4:**
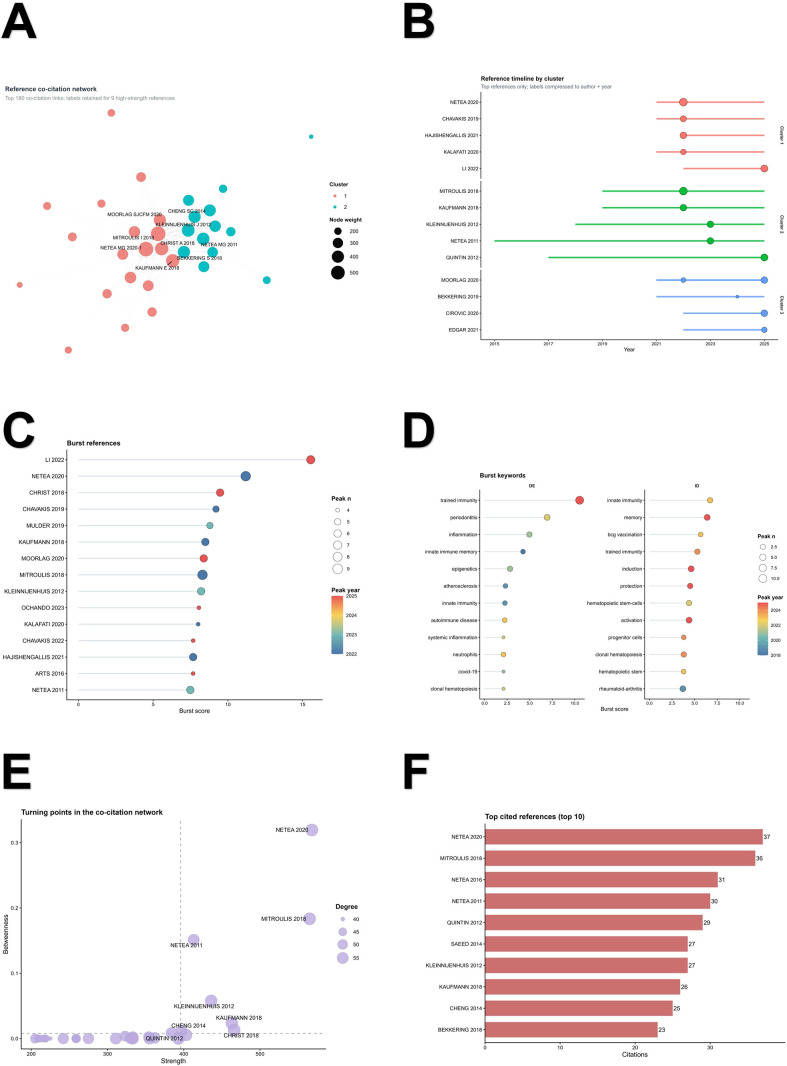
Intellectual foundations and citation dynamics of the field. **(A)** Reference co-citation network displayed as a thresholded high-weight network. To improve readability, the visualized network was restricted to the strongest co-citation links, and labels were retained only for high-strength references. Node size reflects co-citation strength, edge thickness reflects co-citation weight, and node color indicates network cluster. **(B)** Reference timeline by cluster, illustrating the temporal distribution of representative co-cited references across major clusters. **(C)** Burst references ranked by burst score, highlighting highly dynamic or frontier references in the field; dot size indicates peak intensity and color indicates peak year. **(D)** Burst keywords, showing rapidly emerging terms in the bibliometric landscape; dot size indicates peak intensity and color indicates peak year. In **(D)** DE denotes Author Keywords and ID denotes Keywords Plus. **(E)** Turning points in the co-citation network, plotted by strength and betweenness centrality to identify structurally important references. **(F)** Top cited references in the core corpus ranked by citation frequency. Detailed network thresholds and full analytical settings are provided in the **Supplementary Methods** and **Supplementary Tables**. BCG, bacillus Calmette–Guérin.

The intellectual base of the field was strongly anchored in core trained-immunity literature. The reference co-citation network contained 60 nodes and 1318 edges, with the most strongly co-cited references being Netea MG 2020, Mitroulis I 2018, Christ A 2018, Kaufmann E 2018, and Kleinnijenhuis J 2012. Timeline analysis identified three prominent clusters: cluster 1 was centered on Netea MG 2020 and spanned 2019–2025, cluster 2 on Mitroulis I 2018 and spanned 2015–2025, and cluster 3 on Cirovic B 2020 and spanned 2019–2025, with the third cluster peaking in 2025. Burst analysis of cited references further highlighted Li XF 2022, Netea MG 2020, Christ A 2018, Chavakis T 2019, and Kaufmann E 2018 as recent or high-intensity burst references. Keyword burst analysis was consistent with this pattern: in Author Keywords, the strongest burst terms were trained immunity, periodontitis, inflammation, innate immune memory, and epigenetics, whereas in Keywords Plus the strongest burst terms included innate immunity, memory, BCG vaccination, trained immunity, induction, and hematopoietic stem-cells. Together, these results suggest that the field is intellectually rooted in core trained-immunity biology while increasingly extending toward inflammatory bone disease, hematopoietic memory, and translational immune modulation (**Figures 4A–F**).

### 3.5 Topic structure and semantic expansion of the extended corpus

**Figure 5:**
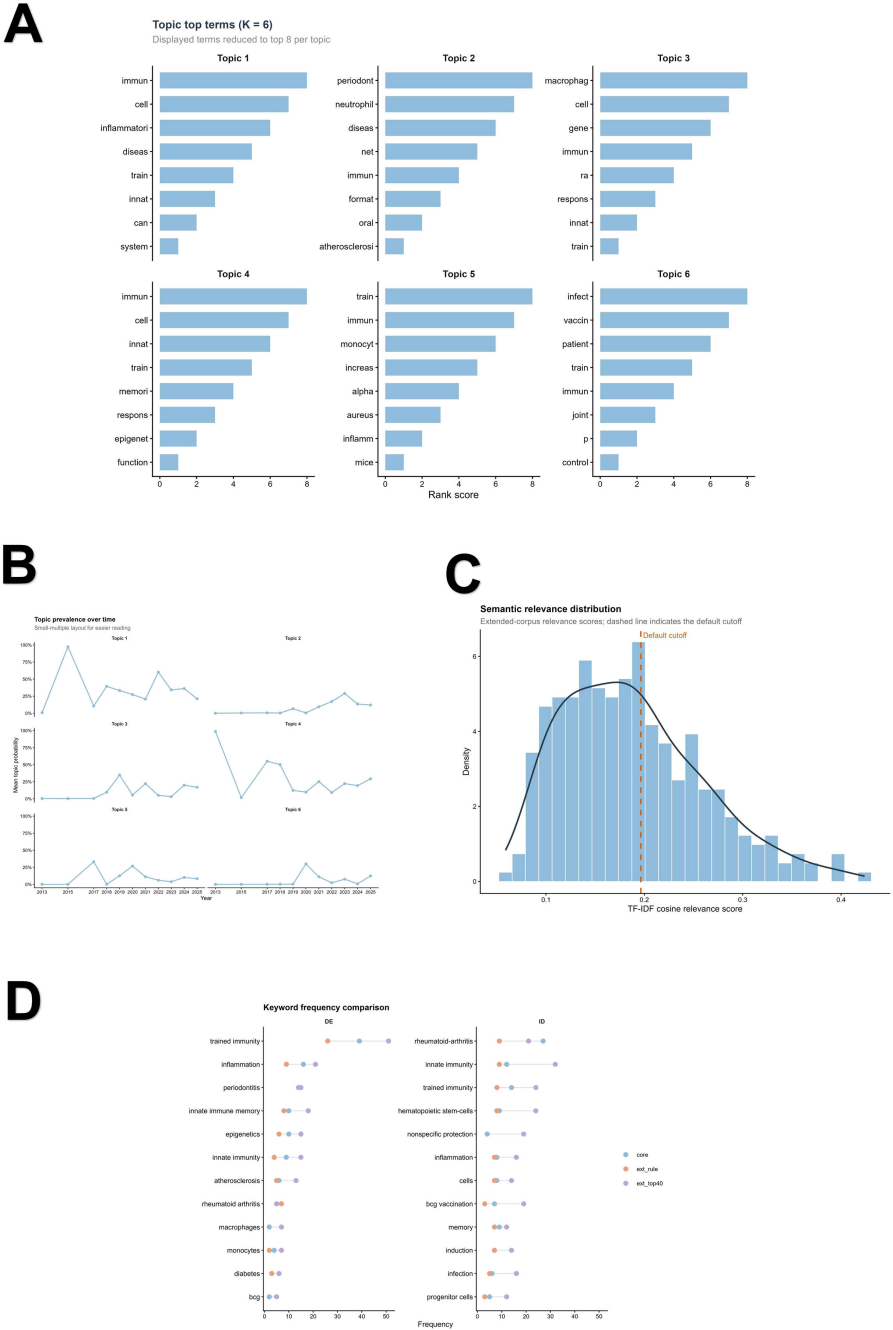
Topic structure and semantic expansion of the field. **(A)** Simplified display of stemmed top terms for the final K = 6 topic solution. To improve readability, the main figure presents the leading terms for each topic, whereas detailed model-selection results, representative documents, and sensitivity analyses are provided in the **Supplementary Methods** and **Supplementary Tables**. **(B)** Topic prevalence over time, showing the temporal distribution of the six topics across publication years. **(C)** Distribution of TF-IDF cosine relevance scores in the extended corpus. The dashed line indicates the cutoff used to define the default semantically relevant subset. **(D)** Keyword frequency comparison among the core corpus, the rule-based subset, and the top-40% semantically selected subset. In the panel legend, “ext_rule” denotes the rule-based subset and “ext_top40” denotes the top-40% semantically selected subset. The K = 6 solution was used as an interpretable topic summary rather than as a uniquely optimal partition of the literature.

Machine-learning-assisted topic modeling of the core corpus was evaluated across neighboring solutions with K = 5–7, and a 6-topic model was retained for the main presentation as an interpretability-based summary of the field. Supplementary LDA-based repeated-run analyses showed that quantitative fit and run-level stability metrics were slightly more favorable for K = 5, whereas K = 6 provided clearer thematic separation with less redundancy in the top-term structure (**Supplementary Methods**; **Supplementary Tables 1–3**). Accordingly, the final topic solution should be interpreted as a pragmatic summary of the literature rather than as a uniquely optimal partition. In the final K = 6 presentation, Topic 1 was characterized by terms related to inflammatory memory and systemic immune responses, Topic 2 by periodontitis-, neutrophil-, and NET-related terms, Topic 3 by macrophage- and arthritis-associated terms, Topic 4 by innate immune memory and epigenetic regulation, Topic 5 by monocyte-centered inflammatory training, and Topic 6 by infection- and vaccination-related content. Temporally, Topic 4 was dominant in 2013, 2017, 2018, 2021, and 2025, whereas Topic 1 dominated in 2015 and again across 2022–2024, suggesting a shift in emphasis from broader innate-memory framing toward disease-linked inflammatory memory and periodontal relevance in later years (**Figures 5A, B**; **Supplementary Methods; Supplementary Tables 1–3**).

Semantic expansion of the extended corpus further refined this topic structure. Relevance screening ranked all 301 extended-corpus documents by the TF-IDF cosine relevance score and retained 121 records (40.2%) as the default semantically relevant subset. Manual cross-checking of the retained subset and cutoff-adjacent records did not reveal material inconsistencies between the automated ranking and title/abstract-level interpretation. The highest-ranked documents were enriched for periodontitis, inflammatory comorbidities, autoimmune and rheumatic disease, and trained-immunity-related therapeutic perspectives. Comparison of keyword distributions between the core corpus, the rule-based subset, and the semantically selected top-40% subset showed strong conceptual continuity together with broader biological reach in the expanded subset. In Author Keywords, the semantically selected subset showed higher frequencies of terms related to trained immunity, inflammation, innate immune memory, epigenetics, atherosclerosis, macrophages, and monocytes. In Keywords Plus, the same subset was additionally enriched for hematopoietic stem cells, BCG vaccination, nonspecific protection, and infection. These results indicate that semantic expansion preserved the core osteoimmunology signal while capturing a broader trained-immunity-related literature periphery relevant to inflammatory bone disease (**Figures 5C, D**).

### 3.6 Entity, disease, and pathway mapping

**Figure 6:**
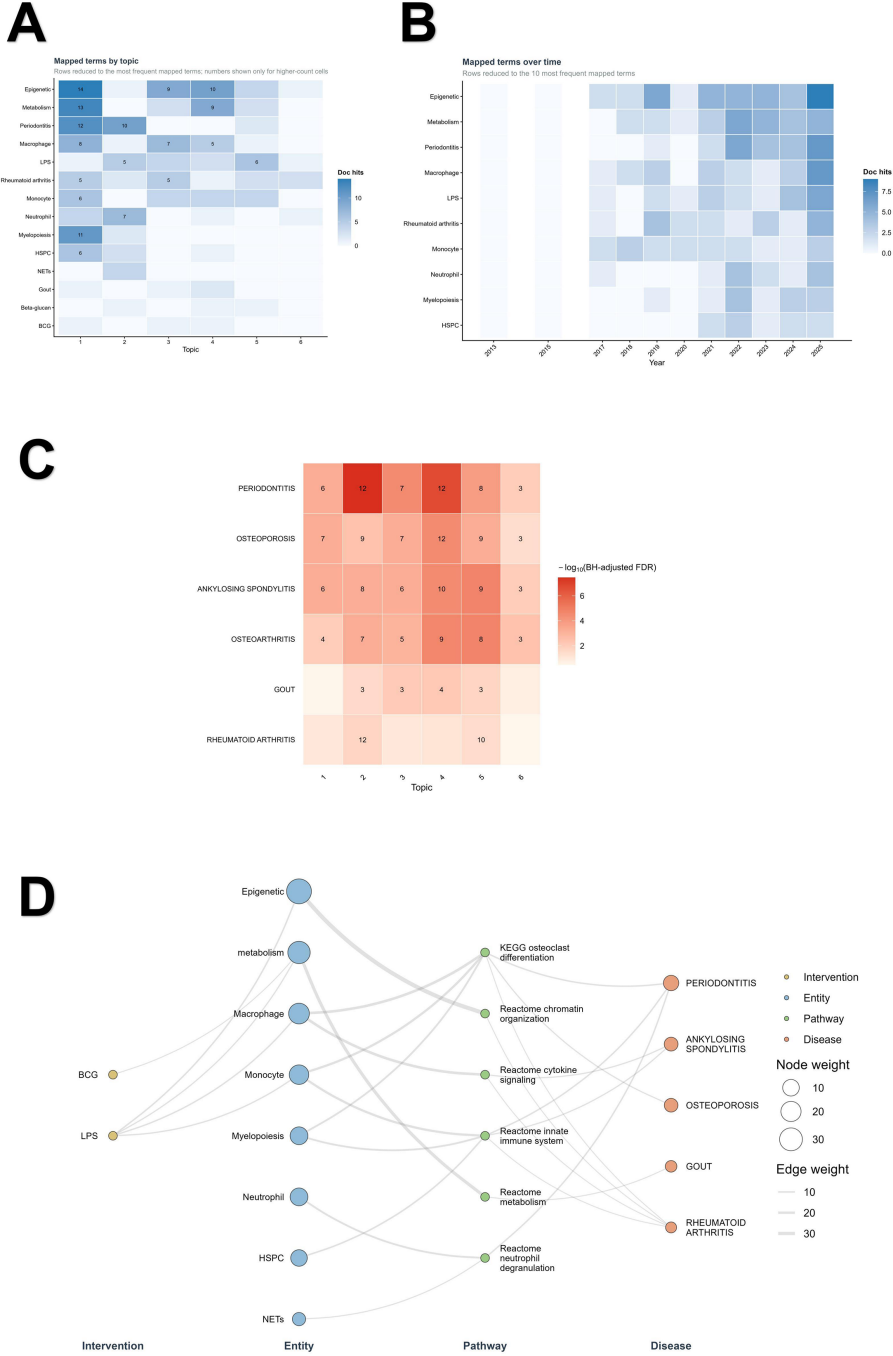
Literature-level entity, disease, and pathway mapping. **(A)** Heatmap of mapped-term hits by topic. To improve readability, the displayed rows were reduced to the most frequent mapped terms, and cell labels were retained only for higher-count cells. **(B)** Heatmap of mapped-term hits by publication year, showing temporal shifts among the most frequent mapped terms. **(C)** Disease-enrichment heatmap across topic clusters. Topic-disease enrichment was evaluated using Fisher’s exact test followed by Benjamini–Hochberg false discovery rate correction. Fill color indicates −log10(FDR), with larger values indicating stronger FDR-corrected enrichment evidence. Cell labels indicate overlap counts for FDR-significant associations. These results represent literature-level disease-context associations rather than evidence of causal disease mechanisms. **(D)** Thresholded four-layer bridge network across intervention, entity, pathway, and disease layers. Node size reflects node weight, edge thickness reflects connection weight, and labels were reduced or shortened to emphasize core high-weight nodes and links. The bridge network summarizes recurrent literature-level connections among trained-immunity-related interventions, mapped biological entities, predefined pathway anchors, and disease contexts. Detailed mapping rules, display thresholds, and interpretation boundaries are provided in the **Supplementary Methods** and **Supplementary Tables**. BCG, bacillus Calmette–Guérin; FDR, false discovery rate; HSPC, hematopoietic stem and progenitor cell; LPS, lipopolysaccharide; NETs, neutrophil extracellular traps.

Multi-layer mapping revealed a consistent mapped-term structure across topics and years. At the topic level, epigenetic- and metabolism-related terms were especially prominent in Topics 1, 3, and 4, whereas Topic 2 was characterized by periodontitis, neutrophil, and LPS, and Topic 5 by LPS, epigenetic, and monocyte. Across the full corpus, the most frequent mapped terms by summed document hits were Epigenetic (39), Metabolism (28), Periodontitis (24), Macrophage (22), LPS (20), Rheumatoid arthritis (19), Monocyte (18), and Myelopoiesis (13). Temporal aggregation suggested that monocyte-related signals appeared earlier, whereas periodontitis-, metabolism-, and epigenetic-related terms became more prominent from 2022 onward. The year 2025 was characterized by particularly strong signals for Epigenetic, Periodontitis, and Macrophage (**Figures 6A, B**; **Supplementary Methods**; **Supplementary Table 4**).

Disease enrichment analysis produced 36 topic-disease association rows, of which 30 remained significant after Benjamini–Hochberg false discovery rate correction. In **Figure 6C**, enrichment intensity is displayed as −log10(FDR), with larger values indicating stronger FDR-corrected enrichment evidence. The strongest enrichment signals were concentrated in periodontitis, osteoarthritis, ankylosing spondylitis, and osteoporosis, with prominent signals across Topics 2, 4, and 5. At a broader level, these findings suggest that the disease-relevant literature space of trained immunity in this field is concentrated not only in periodontal inflammation but also in inflammatory arthritis and bone-remodeling disorders.

The final thresholded bridge network provided an integrative view across intervention, entity, pathway, and disease layers. This network contained 21 nodes and 27 edges, including 8 entity nodes, 6 pathway nodes, 5 disease nodes, and 2 intervention nodes. The highest-weight nodes were Epigenetic (39), Metabolism (28), Macrophage (22), Monocyte (18), Neutrophil (13), and Myelopoiesis (13), indicating that epigenetic, metabolic, and myeloid-associated terms were recurrently prominent in the mapped literature. At the edge level, strong links connected Epigenetic to Reactome Chromatin organization, Metabolism to Reactome Metabolism, Macrophage to Reactome Cytokine signaling in immune system and KEGG Osteoclast differentiation, and Monocyte and Myelopoiesis to Reactome Innate immune system and KEGG Osteoclast differentiation. Additional links connected LPS to Epigenetic and osteoclast differentiation to Periodontitis. Together, these results provide a literature-level map linking epigenetic and metabolic reprogramming, myeloid-associated terms, innate immune signaling, osteoclast differentiation, and osteoimmunology-relevant disease contexts within the thresholded network view (**Figure 6D**).

## 4 Discussion

This study provides a structured literature-level map of trained-immunity-related concepts in osteoimmunology and inflammatory bone disease. The analysis highlights a coherent pattern in which innate immune memory is increasingly linked to myeloid-cell reprogramming, epigenetic and metabolic remodeling, myelopoiesis, osteoclast differentiation, and chronic inflammatory bone loss (18). Recent work on damage-associated molecular pattern (DAMP)-driven trained immunity and chronic inflammatory reprogramming further supports the relevance of metabolic and epigenetic remodeling in persistent innate immune responses (19, 20). Together, these signals bring together disease literatures that are often discussed separately, including periodontal inflammation, inflammatory arthritis, degenerative joint disease, osteoporosis, and infection-related bone pathology.

Periodontitis was the clearest disease-level signal. This finding is biologically plausible because periodontitis combines persistent microbial stimulation, dysregulated neutrophil and monocyte/macrophage responses, cytokine-driven tissue destruction, and alveolar bone loss. Recent work on periodontal inflammation and bone loss emphasizes that bone-resorptive signaling pathways remain difficult to translate into effective clinical interventions, despite increasing mechanistic insight (21). This makes periodontitis a useful disease context for studying whether repeated microbial and inflammatory stimulation can leave durable imprints on innate immune cells, osteoclast precursors, or bone marrow-derived myeloid compartments. The relevance of this direction is further supported by studies linking trained-immunity-related mechanisms, inflammatory comorbidities, and monocyte reprogramming to periodontitis biology (10, 11, 22). Together, these observations support considering periodontitis as a local inflammatory bone disease with potential systemic inflammatory links, rather than as an isolated oral-disease context.

The disease pattern was broader than periodontitis. Osteoarthritis, ankylosing spondylitis, rheumatoid arthritis, osteoporosis, and bone and joint infection also appeared in the mapped literature. These conditions differ in triggers, tissues, immune-cell composition, and remodeling phenotypes, so the shared signal should be interpreted as conceptual convergence rather than as a single common mechanism. Its value lies in showing that trained immunity is becoming a useful language for inflammatory bone disorders in which chronic inflammation, myeloid activation, and pathological remodeling intersect. This interpretation is consistent with recent work emphasizing immune contributions to osteoarthritis (13), inflammatory–osteogenic crosstalk and biomarker discovery in ankylosing spondylitis (14, 23), and immune-cell regulation of osteoporosis (15, 24).

The repeated appearance of epigenetic, metabolic, and myeloid-associated terms helps explain why trained immunity is relevant to this field. Current models describe trained immunity as a long-lasting functional adaptation of innate immune cells driven by epigenetic and metabolic remodeling (18–20). Recent work has also extended this concept to hematopoietic stem and progenitor cells, suggesting that inflammatory exposures may durably shape myelopoiesis and downstream myeloid populations (5, 6). This is particularly relevant to inflammatory bone disease because osteoclasts derive from the monocyte/macrophage lineage, and bone marrow niches participate in both immune regulation and skeletal remodeling. The convergence of monocytes, macrophages, myelopoiesis, metabolism, chromatin organization, and osteoclast differentiation therefore identifies a plausible biological axis for future experimental work.

Recent experimental evidence gives this axis greater weight. Haacke et al. showed that β-glucan-induced trained immunity can promote an osteoclastogenic bias in bone marrow and peripheral osteoclast precursor populations and aggravate inflammatory bone loss in mouse models of periodontitis and arthritis (8). This study provides a strong mechanistic anchor for the field because it connects trained myeloid responses to osteoclastogenesis and skeletal pathology. Other recent studies on inflammatory bone loss also underscore the importance of osteoclast-regulatory pathways, mechanosensing, and immune–bone signaling in periodontitis and related osteolytic conditions (9, 25). Together, these studies support the biological plausibility of the literature-level patterns observed here, while leaving important questions about disease specificity, human validation, and therapeutic timing.

The clinical relevance of these findings lies in the possibility that trained-immunity-associated programs may contribute to disease persistence, flare susceptibility, progressive bone loss, or variable responses to anti-inflammatory treatment. Future studies could test whether circulating monocytes, bone marrow progenitors, or osteoclast precursors from patients with periodontitis, inflammatory arthritis, osteoporosis, or bone and joint infection carry stable epigenetic, metabolic, or transcriptional signatures of innate immune memory. Longitudinal cohorts would be especially useful for determining whether these signatures predict bone-loss progression, inflammatory recurrence, treatment response, or systemic comorbidity. A practical next step is to develop disease-specific biomarkers of innate immune memory and to validate these signals in patient samples, mechanistic models, and translational studies. Broader bone-regeneration and osteoimmune research also supports the importance of immune–stem cell crosstalk and immune-cell communication as translational interfaces linking inflammation and skeletal repair (26, 27).

Therapeutic translation will require caution. Trained immunity can support host defense and tissue repair in some contexts, while amplifying chronic inflammation in others. Recent reviews emphasize this context-dependent duality and the risks of broad immune modulation, especially when trained immunity is considered as a therapeutic target (28, 29). In inflammatory bone disease, the timing, tissue compartment, cellular target, and disease stage are likely to be critical. Strategies that dampen maladaptive myeloid training might reduce inflammatory osteoclastogenesis, but they could also compromise antimicrobial defense or repair responses. Conversely, approaches that enhance innate immune readiness could be harmful in diseases dominated by chronic inflammatory remodeling. Evidence-synthesis frameworks in other immune-mediated inflammatory diseases illustrate the level of validation required before therapeutic claims can be made, but inflammatory bone disease has not yet reached that stage for trained-immunity-directed interventions (30).

The intellectual structure identified in this study is consistent with both foundational and recent trained-immunity literature highlighted by the co-citation analyses. Core and updated references in the field have established trained immunity as a durable innate immune program, linked it to myelopoiesis and progenitor-cell adaptation, and defined metabolic and epigenetic remodeling as central mechanisms (31–34). Together, these studies further connect trained immunity to hematopoietic stem and progenitor cell programming, monocyte/macrophage functional reprogramming, glycolytic remodeling, histone lactylation, and epigenetic memory (35–38). These foundational and recent updates help explain why the present literature map repeatedly converged on myeloid memory, metabolism, chromatin organization, and osteoclast-related pathways.

The two-corpus design helps place these findings in context. The core corpus preserved disease specificity, while the extended corpus showed that the focused osteoimmunology literature is connected to a wider trained-immunity landscape involving infection biology, vaccination, immunometabolism, hematopoietic memory, and inflammatory comorbidity research. The semantic subset should be read as a boundary-assessment tool, not as a rigid classification of relevant and irrelevant studies. This distinction is useful for readers because it separates the disease-focused core from the broader conceptual periphery and clarifies where future studies may bridge currently separated literatures.

Recent reviews of trained immunity in chronic inflammatory disease and immune modulation further reinforce the need for disease-specific interpretation (39, 40). In parallel, emerging osteoimmunology work on extracellular vesicles and aging-related immune–bone interactions suggest that inflammatory bone disease may involve multiple layers of immune communication beyond classical cytokine signaling alone (41, 42). These developments support a focused message: trained immunity is emerging as a useful framework for organizing inflammatory bone disease research, especially where myeloid memory, osteoclastogenesis, and chronic inflammatory remodeling intersect. The present analysis identifies the most visible disease contexts and biological themes in the literature, while also defining the boundaries of current evidence. The next stage of the field should move from literature-level convergence toward disease-specific mechanistic testing, patient-level validation, and clinically interpretable biomarkers.

## 5 Limitations

Several limitations should be acknowledged. First, WoSCC was used as the primary corpus for formal bibliometric analyses, whereas Scopus and PubMed were used for independent cross-database comparison rather than for construction of a merged master corpus. This design improved citation-data consistency and avoided instability from merging databases with different metadata structures. However, database-specific coverage, indexing practices, update schedules, and metadata completeness may still have influenced the observed bibliometric landscape. Although duplicate checks, DOI normalization, field harmonization, and independent cross-database comparisons were used to reduce retrieval and metadata-related bias, these procedures cannot fully eliminate database-specific retrieval bias.

Second, the search strategy had to balance specificity and breadth. The core corpus was intentionally focused, which improved disease relevance but limited the scale of some bibliometric inferences. A narrower strategy could miss peripheral studies relevant to innate immune memory and bone remodeling, whereas a broader strategy could introduce records only indirectly related to inflammatory bone disease. The inclusion of both Articles and Reviews may also have influenced thematic patterns because review articles can amplify interpretive concepts even when primary experimental evidence remains limited.

Third, topic modeling, semantic screening, entity extraction, disease enrichment, and bridge-network construction depend on preprocessing rules, dictionaries, corpus boundaries, and threshold settings. We addressed this by evaluating neighboring topic solutions, examining alternative semantic cutoffs, applying an independent rule-based subset, and manually checking the default semantic subset and cutoff-adjacent records at the title/abstract level. These procedures support the robustness of the main interpretation, although they do not remove the inherent subjectivity of literature-level text-mining workflows.

Finally, the entity-to-pathway and disease-context mappings were derived primarily from titles, abstracts, keywords, and curated-resource alignment rather than from direct experimental measurements. They should therefore be interpreted as exploratory and hypothesis-generating summaries. They do not establish causal pathway activation, disease mechanisms, or therapeutic efficacy in specific inflammatory bone disease models.

## 6 Conclusion

In conclusion, research on trained immunity in osteoimmunology and inflammatory bone loss remains relatively compact but has expanded steadily and now shows a recognizable thematic and intellectual structure. The field is anchored in core trained-immunity biology and is increasingly extending to disease contexts such as periodontitis, osteoarthritis, ankylosing spondylitis, and osteoporosis. Across the bibliometric, semantic, and mapping analyses, recurrent literature-level patterns involved epigenetic remodeling, immunometabolism, macrophage- and monocyte-associated reprogramming, innate immune signaling, and osteoclast-related pathways. Together, these findings provide a structured literature-level overview of an emerging field and may help guide future mechanistic, experimental, and translational work.

## Data Availability

The original contributions presented in the study are included in the article/**Supplementary Material**. Further inquiries can be directed to the corresponding authors.
