# Supplementary material for: Trained immunity in inflammatory bone disease: a bibliometric and literature-level text-mining analysis
Source: Front Immunol. 2026 May 20;17:1832996. doi: 10.3389/fimmu.2026.1832996 (PMC13229834; doi:10.3389/fimmu.2026.1832996)
Supplement: Supplementary file 1 [file DataSheet1.pdf]

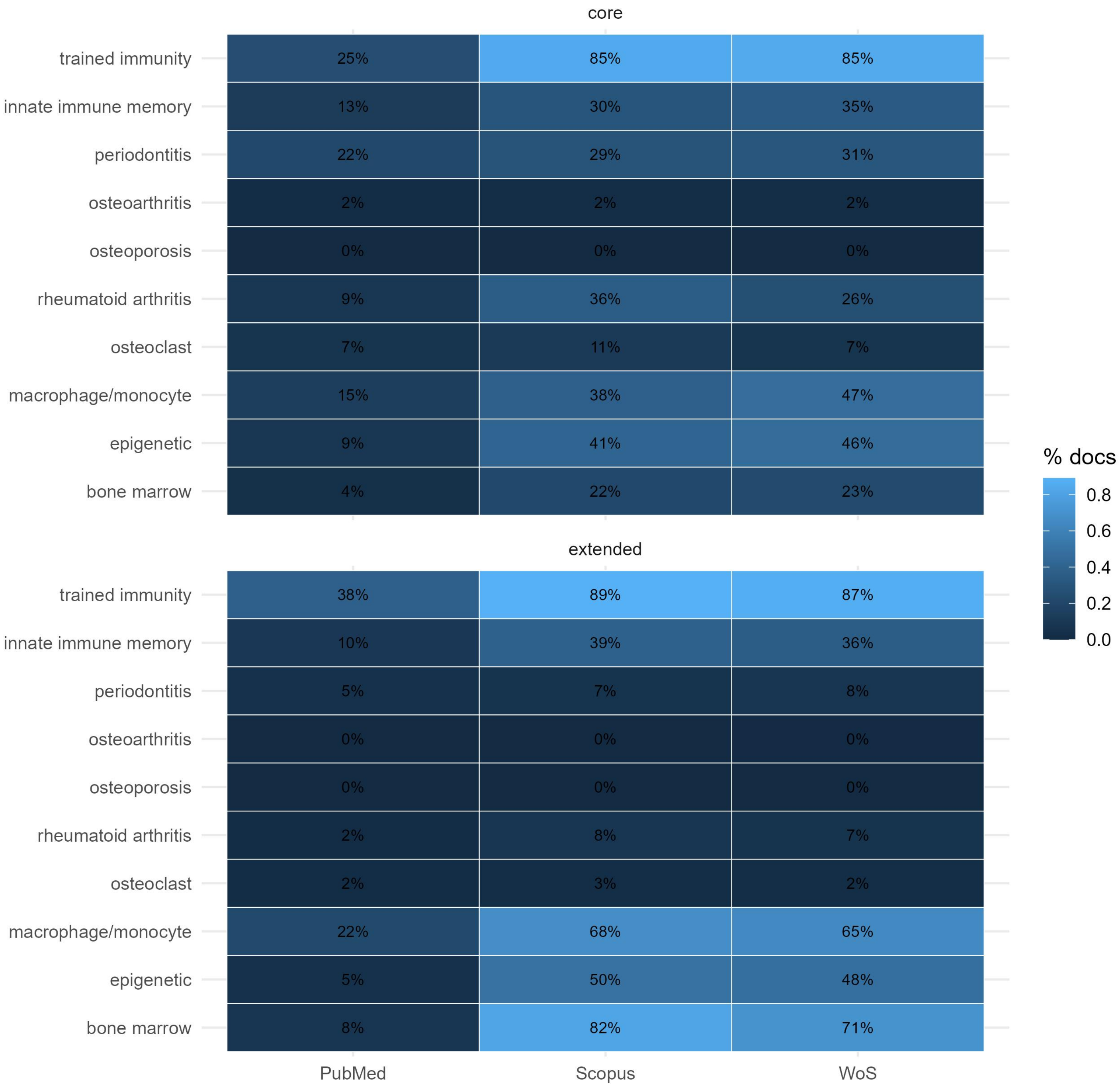

Supplementary Figure S1. Cross-database thematic concordance for selected anchor terms.

Heatmap showing the proportions of documents containing selected anchor terms across WoSCC, Scopus, and PubMed query sets within the 2013–2025 comparison window. Results are shown separately for the core and extended query layers. Anchor-term matching was based on harmonized text fields derived from titles, abstracts, and available keyword fields after database-specific field normalization. Cell values indicate the proportion of records in each database-specific query set containing the corresponding anchor term; the corresponding frequencies and proportions are provided in Supplementary Table S8.
