# Supplementary material for: Trained immunity in inflammatory bone disease: a bibliometric and literature-level text-mining analysis
Source: Front Immunol. 2026 May 20;17:1832996. doi: 10.3389/fimmu.2026.1832996 (PMC13229834; doi:10.3389/fimmu.2026.1832996)
Supplement: Supplementary file 2 [file Table1.docx]

**Supplementary Methods**

**Analytical parameters and mapping rules**

**Overview**

The main Methods summarize the study design and analytical workflow. The present Supplementary Methods provide software versions, preprocessing thresholds, model settings, semantic-screening rules, mapping dictionaries, and interpretation boundaries used to support reproducibility.

All analyses were conducted in R version 4.5.1. Bibliometric workflows were implemented primarily using bibliometrix/Biblioshiny version 5.2.1. Text preprocessing and document-feature workflows were implemented using quanteda version 4.3.1 and text2vec version 0.6.6. The primary topic-model presentation was generated using stm version 1.3.8. Supplementary repeated-run topic-model checks were performed using topicmodels version 0.2.17.

**Search dates and retrieval records**

The final WoSCC searches were conducted on 2 March 2026. The final Scopus and PubMed searches were conducted on 17 March 2026. Records were exported on the corresponding retrieval dates. Complete database-specific search strings, filters, retrieval dates, and corpus roles are provided in Supplementary Table S5, and database-specific retrieval, filtering, and retained comparison-set counts are summarized in Supplementary Table S6. For PubMed, the language and publication-type restrictions were embedded in the final query, so a separate unfiltered raw pre-filter count was not recorded.

**Corpus construction and filtering**

WoSCC records served as the primary input for formal bibliometric and text-mining analyses. Only English-language publications indexed as Article or Review were retained. Under the core search strategy, 88 records were retrieved and 83 were retained after restriction to Article and Review document types. Under the extended search strategy, 322 records were retrieved and 301 were retained after exclusion of 2 non-English records and 19 records not indexed as Article or Review. These retained records constituted the full WoS primary datasets.

Scopus and PubMed were queried independently using database-adapted versions of the two-layer search design. These records were used for cross-database comparison rather than for construction of a merged master corpus. For this comparison, database-specific query sets were harmonized under equivalent language and document-type logic and restricted to publications from 2013 to 2025. Within this window, the retained query sets comprised 81 WoS core records, 92 Scopus core records, 55 PubMed core records, 292 WoS extended records, 384 Scopus extended records, and 264 PubMed extended records. Database-specific retrieval yields, filtered counts, and retained query sets are summarized in Supplementary Table S6.

**Cross-database comparison**

Cross-database comparison focused on query-set size, annual publication trends, DOI-level overlap, and thematic concordance. DOI strings were normalized before overlap analysis, and database fields were harmonized to compare query-set characteristics across WoSCC, Scopus, and PubMed.

Detailed DOI-level overlap statistics are reported in Supplementary Table S7. The anchor-term summary underlying thematic concordance is provided in Supplementary Table S8. Exact equality of raw record counts was not expected because the databases differ in coverage, indexing practices, field structures, and update schedules.

**Text preprocessing**

For topic modeling and semantic analyses, titles and abstracts were converted to lowercase. Punctuation and numbers were removed. English stopwords were removed, and tokens were stemmed. Generic non-informative terms were removed using a curated stoplist. Synonym harmonization was applied to trained-immunity-related terms, disease names, institution names, and country names.

In the main STM workflow, document-feature trimming used min_termfreq = 3. In the supplementary LDA-based checks, preprocessing thresholds were min_doc_term_freq = 2, min_df_prop = 0.02, and max_df_prop = 0.95.

**Bibliometric analyses and visualization settings**

Bibliometric analyses were conducted using bibliometrix/Biblioshiny-based workflows. The analyses summarized annual publication output, journals, authors, institutions, countries, Author Keywords, Keywords Plus, co-authorship networks, keyword co-occurrence structures, thematic maps, reference co-citation networks, and burst patterns.

Author Keywords and Keywords Plus were analyzed separately. Author Keywords were treated as author-defined descriptors, whereas Keywords Plus were treated as database-generated indexing terms. Co-authorship analyses were performed at the author, institution, and country levels. Country-level full-counting results were reported separately from article-level single-country publication and multiple-country publication summaries.

Temporal bibliometric analyses were performed at annual resolution. Because 2026 was incomplete at retrieval, year-based cross-database comparisons were restricted to 2013–2025.

**Primary topic-model presentation**

The main topic-model presentation was generated using the stm framework. Candidate solutions with K = 5–7 were evaluated. Five STM runs were performed for each K value, with seeds defined as 2000 + 10 × K + r, where r denotes the run index. STM models were fitted using init.type = "Spectral" and max.em.its = 75.

The final displayed model retained K = 6 because it provided clearer thematic separation than K = 5 while avoiding greater redundancy and fragmentation than K = 7. Final selection was guided by interpretability together with semantic coherence and exclusivity. The K = 6 solution was interpreted as a pragmatic summary of recurring thematic structure rather than as a uniquely optimal partition of the literature.

**Supplementary topic-model checks**

Supplementary repeated-run checks were performed using topicmodels::LDA with variational EM across K = 5–7. These analyses used 20 runs per K, with seeds defined as 1000 + k × 100 + i, where i denotes the run index.

Run-level outputs were summarized using log-likelihood, perplexity, topic cosine similarity, top-term Jaccard overlap, document-assignment agreement, and document-topic concentration metrics. These outputs are reported in Supplementary Tables S1–S3. They were used to contextualize neighboring topic solutions and assess run-level consistency, not to replace the primary STM-based presentation.

**Semantic relevance scoring and corpus-boundary analyses**

For the extended corpus, semantic relevance to the core corpus was quantified using a TF-IDF cosine relevance score. A centroid representation was first calculated for the core corpus. Each extended-corpus record was then represented in the same TF-IDF feature space and ranked by cosine similarity to the core-corpus centroid.

The default semantically relevant subset retained the top 40% of ranked records, corresponding to 121 of 301 records. Additional sensitivity analyses examined 30%, 40%, and 50% cutoffs.

An independent rule-based subset was also defined using predefined osteoimmunology anchor terms: joint, synovial-related terms, osteoclast, bone loss, erosion, periodontitis, and rheumatoid arthritis (RA). The rule-based subset contained 53 records, and the overlap between the rule-based subset and the top-40% semantic subset was 45 records.

Comparative keyword profiling and overlap across these subsets were used as boundary checks for corpus interpretation and corpus-definition bias. The default top-40% subset and cutoff-adjacent records were manually reviewed at the title/abstract level to assess whether the automated ranking introduced material interpretation-level discordance.

**Multi-layer mapping framework**

A four-layer mapping framework was applied to titles, abstracts, and keywords. The predefined layers were intervention/stimulus, entity, pathway, and disease context. Cell types, cellular programs, molecular processes, stimuli/interventions, and disease terms were harmonized through synonym normalization, regex-based dictionary matching, and rule-based aggregation.

Mapped-term profiles were summarized at the document, year, and topic levels. These summaries were used to characterize recurring literature-level signals and to support biological interpretation.

**Disease-context mapping**

For disease-context interpretation, topic-associated terms were linked to DisGeNET Gene-Disease Associations through the Harmonizome API. Topic-disease enrichment was evaluated using Fisher’s exact test, and multiple-testing correction was performed using the Benjamini–Hochberg false discovery rate procedure. The disease-enrichment heatmap displays −log10(FDR), where larger values indicate stronger FDR-corrected enrichment evidence. Cell labels indicate overlap counts for FDR-significant associations. These enrichment results were interpreted as literature-level disease-context associations rather than as evidence of causal disease mechanisms.

**Pathway-context mapping and bridge-network construction**

Bridge-network construction was anchored to a predefined set of osteoimmunology-relevant pathways. These anchors included KEGG:hsa04380 Osteoclast differentiation and selected Reactome pathways related to innate immune system, cytokine signaling in immune system, neutrophil degranulation, chromatin organization, and metabolism.

The final bridge network was displayed as a thresholded four-layer network spanning intervention, entity, pathway, and disease layers. Node weights reflected mapped-term prominence, and edge weights reflected co-occurrence or dictionary-supported links under the predefined mapping framework. The display was intended to summarize recurring literature-level connections among trained-immunity-related interventions, biological entities, pathway anchors, and disease contexts.

**Interpretation boundary**

All topic-model, semantic relevance, disease-mapping, and bridge-network outputs were designed to support literature-level interpretation. These outputs were derived from titles, abstracts, keywords, and curated-resource relationships rather than from direct experimental data.

Accordingly, the analyses were interpreted as exploratory and hypothesis-generating. They were not used to infer causal mechanisms, estimate intervention effects, or establish pathway activation in specific disease models.
